# Selective stimulation of colonic L cells improves metabolic outcomes in mice

**DOI:** 10.1007/s00125-020-05149-w

**Published:** 2020-04-27

**Authors:** Jo E. Lewis, Emily L. Miedzybrodzka, Rachel E. Foreman, Orla R. M. Woodward, Richard G. Kay, Deborah A. Goldspink, Fiona M. Gribble, Frank Reimann

**Affiliations:** Wellcome Trust-MRC Institute of Metabolic Science-Metabolic Research Laboratories, University of Cambridge, Cambridge, CB2 OQQ UK

**Keywords:** Colonic L cells, Enteroendocrine cells, GLP-1, Glucagon-like peptide-1, INSL5, Insulin-like peptide-5, Peptide YY, PYY

## Abstract

**Aims/hypothesis:**

Insulin-like peptide-5 (INSL5) is found only in distal colonic L cells, which co-express glucagon-like peptide-1 (GLP-1) and peptide YY (PYY). GLP-1 is a well-known insulin secretagogue, and GLP-1 and PYY are anorexigenic, whereas INSL5 is considered orexigenic. We aimed to clarify the metabolic impact of selective stimulation of distal colonic L cells in mice.

**Methods:**

*Insl5* promoter-driven expression of Gq-coupled Designer Receptor Exclusively Activated by Designer Drugs (DREADD) was employed to activate distal colonic L cells (L^distalDq^). IPGTT and food intake were assessed with and without DREADD activation.

**Results:**

L^distalDq^ cell stimulation with clozapine *N*-oxide (CNO; 0.3 mg/kg i.p.) increased plasma GLP-1 and PYY (2.67- and 3.31-fold, respectively); INSL5 was not measurable in plasma but was co-secreted with GLP-1 and PYY in vitro. IPGTT (2 g/kg body weight) revealed significantly improved glucose tolerance following CNO injection. CNO-treated mice also exhibited reduced food intake and body weight after 24 h, and increased defecation, the latter being sensitive to 5-hydroxytryptamine (5-HT) receptor 3 inhibition. Pre-treatment with a GLP1 receptor-blocking antibody neutralised the CNO-dependent improvement in glucose tolerance but did not affect the reduction in food intake, and an independent group of animals pair-fed to the CNO-treatment group demonstrated attenuated weight loss. Pre-treatment with JNJ-31020028, a neuropeptide Y receptor type 2 antagonist, abolished the CNO-dependent effect on food intake. Assessment of whole body physiology in metabolic cages revealed L^distalDq^ cell stimulation increased energy expenditure and increased activity. Acute CNO-induced food intake and glucose homeostasis outcomes were maintained after 2 weeks on a high-fat diet.

**Conclusions/interpretation:**

This proof-of-concept study demonstrates that selective distal colonic L cell stimulation has beneficial metabolic outcomes.

**Figure:**
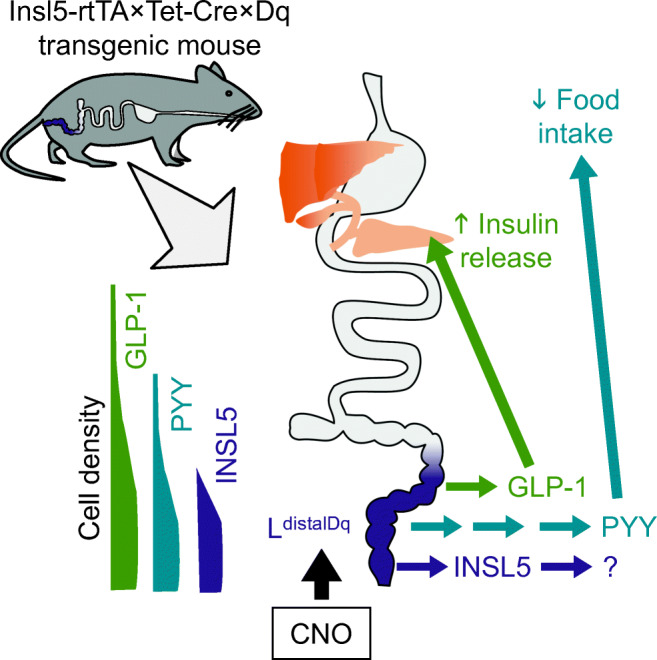
Graphical abstract

**Electronic supplementary material:**

The online version of this article (10.1007/s00125-020-05149-w) contains peer-reviewed but unedited supplementary material, which is available to authorised users.

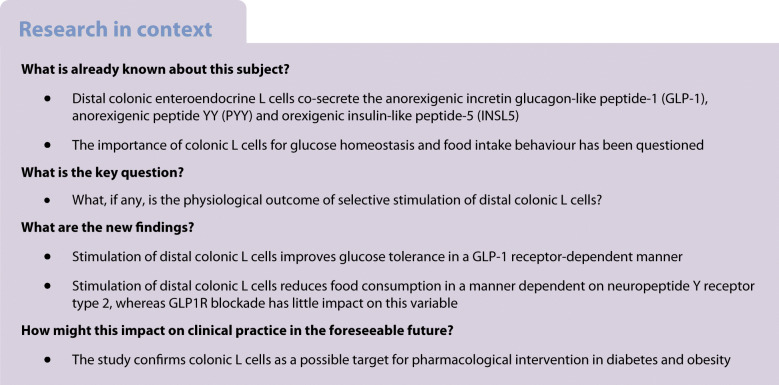



## Introduction

Enteroendocrine cells (EECs) are specialised gastrointestinal epithelial cells that regulate physiological processes ranging from intestinal motility and secretion to glucose homeostasis and appetite [1]. Glucagon-like peptide (GLP)-1, generated from the gene encoding proglucagon (*Gcg*) in intestinal L cells, has both insulinotropic and anorexigenic activities, underlying the clinical use of GLP-1 mimetics for the treatment of type 2 diabetes and obesity [2]. It is produced alongside another proglucagon product, GLP-2, in increasing amounts in the distal gut, where it is co-stored and released with the anorexigenic hormone peptide YY (PYY). In the distal colon and rectum, L cells also produce insulin-like peptide-5 (INSL5), a hormone with reported orexigenic activity [3], and GLP-1, PYY and INSL5 are co-stored and co-released from an overlapping vesicular pool in these cells [4]. It remains unclear, however, whether the physiological roles of colonic L cells are restricted to the local control of motility and maintenance of epithelial integrity, or whether they are also metabolically relevant for the control of glucose homeostasis and appetite.

Secretion of most gut hormones is triggered by ingested nutrients, which activate L cells via G protein-coupled receptors and glucose transporters during their absorption across the epithelium. As most nutrient absorption occurs in the proximal small intestine, it is broadly accepted that EECs in the upper gut underlie the elevated plasma levels of GLP-1 and glucose-dependent insulinotropic peptide (GIP) observed early after meal ingestion and the stimulation of insulin secretion via the incretin effect. The physiological roles of L cells located more distally are less clear. GLP-1 and PYY from L cells in the lower small intestine have been implicated in the ileal brake, responsible for slowing gastric emptying when the rate of nutrient delivery into the duodenum exceeds its capacity for digestion and absorption. Only under exceptional conditions, or long after a meal, do ingested nutrients reach as far as the colon, and it therefore seems unlikely that colonic GLP-1 and PYY contribute to the incretin effect or early postprandial control of appetite [5]. Nevertheless, colonic L cells recapitulate the responsiveness of L cells from more proximal regions, secreting GLP-1 in response to a range of nutrient-related stimuli, including glucose in primary cultures [6–8] and perfused colon preparations [9]. L cells in the colon and rectum also express G-protein-coupled receptors for short-chain fatty acids (SCFAs) [10] generated by the intestinal microbiome and for bile acids [11], which are incompletely absorbed in the distal ileum and modified by luminal bacteria, although the physiological importance of SCFAs for the regulation of GLP-1 release remains uncertain [12]. They also express receptors for angiotensin and arginine vasopressin, leading to suggestions that the physiological roles of L cells in the distal gut may involve regulation of local processes such as fluid secretion, motility and intestinal repair [13, 14].

Although the importance of intestinal GLP-1 for the incretin effect has been questioned [15, 16], deletion of *Gcg* from the gut epithelium in mice lowered circulating active GLP-1 levels and impaired oral glucose tolerance [17]. When *Gcg* was deleted only from the ileum and colon, active GLP-1 levels were reduced during fasting but not after an oral glucose challenge, suggesting a greater contribution of the distal gut to basal than postprandial GLP-1 release [17]. Not only does the relevance of GLP-1 secretion from distal L cells for glucose homeostasis therefore still remain uncertain, but it is also puzzling that L cells in the colon and rectum should co-release two anorexigenic hormones (GLP-1, PYY) together with a reportedly orexigenic peptide (INSL5). Understanding the metabolic role of colonic L cells is particularly relevant because the distal gut harbours the majority of endogenous GLP-1 and PYY stores and recruiting this L cell population could be developed as a therapeutic strategy for diabetes and obesity, provided the hormones exhibit metabolic activity when released from this region. That GLP-1 and PYY from the colon would retain metabolic bioactivity is not necessarily a given, first because GLP-1 is rapidly inactivated in the circulation by dipeptidyl peptidase-4, and second because any activity of EEC-derived peptides on local nerve endings could have different effects depending on local innervation patterns.

To assess the metabolic importance of distal colonic L cells we developed a new mouse model in which tetracycline (doxycycline, DOX)-inducible Cre-mediated recombination results in expression of Dq-Designer Receptors Exclusively Activated by Designer Drugs (DREADD) only in distal colonic (INSL5^+^) L cells (L^distalDq^). We assessed whether selective stimulation of these cells by clozapine *N*-oxide (CNO) can modulate insulin secretion, glucose handling and feeding behaviour and used pharmacological tools to separate the contribution of co-secreted hormones to whole body metabolism.

## Methods

### Animals

Adult male and female mice (aged 3–6 months) were obtained from colonies maintained at the University of Cambridge under specific-pathogen-free conditions, group housed whenever possible. All animal procedures were approved by the University of Cambridge Animal Welfare and Ethical Review Body and carried out in accordance with the Animals (Scientific Procedures) Act 1986. The work was performed under the UK Home Office project licences 70/7824 and PE50F6065. Mice were housed in individual ventilated cages on a 12 h light/dark cycle (lights out at 07:00 GMT) with unrestricted access to water and regular chow (unless otherwise stated). Mice were allowed to acclimatise to the procedural room 1 h prior to intervention. Treatment order was randomised (via GraphPad Prism, version 7.9, GraphPad, USA). Animal experimenters were not blinded to treatment; however, hormone assessments were performed by other staff blinded to the treatment. Mice were culled by an approved Schedule 1 method for tissue collection.

### Mice strains

Generation of the tetracycline-dependent expression of reporter genes in *Insl5*-expressing cells has been previously described [4]. Insl5-rtTA×GCaMP6fΔCMV mice [4] were used to explore *Insl5* promoter driven reporter gene expression in the CNS. To selectively activate *Insl5*-expressing cells through a designer receptor approach, Insl5-rtTA mice were crossed with Tet-Cre- and CAG-Dq DREADD Cre-reporter mice (stock numbers 006234 and 026220, respectively, Jackson Laboratory, USA) to create a triple transgenic model (Insl5-rtTA×Tet-Cre×Dq). All models were backcrossed for more than eight generations onto a mixed C57BL/6J – C57BL/6N background and mice of the same background (C57BL/6JN), but negative for Insl5-rtTA, Tet-Cre and CAG-Dq were used as controls.

### Transgenic expression and stimulation

Mice were treated with DOX in the drinking water (3 mg/ml, with 5% [wt/vol.] sucrose) for a minimum of 5 days prior to intervention. Mice received either vehicle (PBS with 0.05% [vol./vol.] DMSO) or CNO (0.3 mg/kg in PBS/DMSO) via i.p. injection. Testing was performed in a randomised crossover design unless otherwise stated.

#### IPGTT

Mice were singly housed and fasted overnight (15 h). At the zero timepoint, glucose was administered at 2 g/kg body weight i.p., and vehicle or CNO (0.3 mg/kg body weight) was delivered i.p. contralaterally. Blood glucose was measured at 0, 15, 30, 60, 90 and 120 min post administration. Animals were re-fed for 1 h before re-housing. A minimum duration of 7 days between testing was employed. For the GLP-1 receptor (GLP1R) antagonism studies, animals received either an isotype control antibody or a GLP1R blocking antibody (GLP1R0017), developed in collaboration with MedImmune/AstraZeneca (Cambridge, UK) [18], at 19.2 mg/kg s.c., 24 h prior to the IPGTT in a non-crossover design, as described previously [19].

#### Food intake

Mice were singly housed and fasted overnight (15 h) and body weight was then measured. Vehicle or CNO was administered i.p. as described above. Animals were re-fed and food intake measured at 1, 2, 4, 6 and 24 h post administration. Body weight was re-measured at 24 h. A minimum duration of 7 days between testing was employed. For the Y2R antagonism studies, animals received an i.p. injection of either vehicle or the neuropeptide Y receptor type 2 (Y2R) antagonist JNJ-31020028 (20 mg/kg), 30 min prior to contralateral administration of vehicle or CNO, and measurement of food intake as described in a non-crossover design. Pair-feeding was achieved by matching the food intake of the treatment group; animals were fed at about 09:00 hours.

#### Metabolic cages

Oxygen consumption ($\dot{V}{\mathrm{O}}_2$), carbon dioxide production ($\dot{V}{\mathrm{CO}}_2$) and locomotor activity (ambulatory beam breaks) were measured concurrently using a modified open-circuit calorimeter (MetaTrace, Ideas Studio, UK). $\dot{V}{\mathrm{O}}_2$ and $\dot{V}{\mathrm{CO}}_2$ were used to calculate the respiratory exchange ratio (RER) and energy expenditure (EE) as previously described [20]. Mice were placed in the metabolic cages 24 h before starting the experiment and fasted for 16 h overnight. Food was reintroduced at time zero, at the start of the recording, when measurements were taken at 12 min intervals.

#### Defecation

Mice were singly housed overnight and transferred to a clean cage prior to testing. One hour after treatment with vehicle or CNO, faecal pellets in the cage were counted and weighed.

#### Transfer to a high-fat diet

Animals previously tested on standard chow were singly housed and transferred to a high-fat diet (HFD; D12451, Open Source Diets, 45% calories from fat, USA). Animals received either vehicle or CNO at time zero. Food intake was measured at 1, 2, 4, 5 and 24 h post-transfer to the HFD. Body weight was measured prior to re-housing and initiation of HFD. HFD and body weight were monitored for 2 weeks before repeating the IPGTT and food intake studies.

#### Plasma glucose and hormones

All blood samples were collected into capillary tubes via the tail veil in free-moving, conscious animals. Samples were placed immediately on ice, blood glucose measured (5 μl plasma, Accu-Chek, UK) and plasma collected post centrifugation and stored at −80°C until required. Circulating hormones were measured via ELISA (MesoScale Discovery, total GLP-1 and PYY assays, UK) at the Core Biochemical Assay Laboratories, Cambridge, UK. Assay plasma volumes were: insulin 5 μl, GLP-1 15 μl, PYY 40 μl.

#### Immunohistochemistry

We prepared 10-μm-thick colonic and pancreatic sections and 25-μm-thick brain sections following fixation in 4% paraformaldehyde (wt/vol.) overnight at 4°C and a sucrose gradient (15% [wt/vol.] for 6 h, 30% overnight) as previously described [4, 21]. Colonic cells positive for INSL5, 5-hydroxytryptamine (5-HT, serotonin), GCG and GFP (for details of antibodies used, see Electronic supplementary material [ESM] Table 1) staining were manually counted and colocalisation assessed using a CellDiscoverer7 (Zeiss, Germany) and imaged using an SP8 confocal microscope (Leica Microsystems, Germany) with a 63× objective lens. Minor alterations were made during the preparation of coronal CNS section from the previously described method. Mice were anaesthetised with Dolethal (Vetoquinol, Towcester, UK) before being transcardially fixated with 4% PFA in PBS, as described previously [21]. Tissue postfixed for 24 h in 4% PFA overnight and a sucrose gradient (15% [wt/vol.] for 6 h, 30% overnight) was sectioned using a freezing sliding microtome. Sections were blocked for 1 h in 5% donkey serum, 0.3% (vol./vol.) Tween-20 in PBS, sequentially incubated with GFP antiserum (1:1000, catalogue no. 5450, Abcam), biotinylated donkey anti-goat IgG (1:400, Millipore) and avidin-biotin complex (Vector Laboratories.) and developed using DAB (Abcam), before being dehydrated with an ethanol gradient and mounted with Pertex mounting medium (Pioneer Research Chemicals, PRC/R/750).

#### Primary cultures

Crypts were isolated from the colon/rectum of mice treated with DOX as previously described [4]. Briefly, tissue was digested with 0.35 mg/ml collagenase type XI (Sigma, USA). Crypts were cultured on 12-well plates pre-coated with 2% (vol./vol.) Matrigel (BD Biosciences, USA) in 25 mmol/l glucose DMEM with 10% (wt/vol.) FBS, 2 mmol/l L-glutamine, 100 U/ml penicillin, 0.1 mg/ml streptomycin and 10 μmol/l Y-27632 dihydrochloride (Tocris, UK), supplemented with DOX (0.5 μg/ml). Cultures (24 h post plating) were washed in warm saline buffer (138 mmol/l NaCl, 4.5 mmol/l KCl, 4.2 mmol/l NaHCO_3_, 1.2 mmol/l NaH_2_PO_4_, 2.6 mmol/l CaCl_2_, 1.2 mmol/l MgCl_2_, 10 mmol/l HEPES; pH 7.4; 1 mmol/l glucose, 0.0005% [wt/vol.] fatty acid-free BSA) for 30 min at 37°C, then incubated for 1 h at 37°C with test agents dissolved in 600 μl saline buffer. Cultures were treated with vehicle (control) or 10 μmol/l CNO, or elevated glucose (10 mM) in combinations with forskolin/IBMX (10 μmol/l each), a positive control for enteroendocrine cell stimulation. Supernatants were centrifuged at 2000*g* for 5 min at 4°C and snap-frozen prior to LC-MS analysis. Cell cultures were lysed (0.0125 g/l deoxycholic acid, 50 mmol/l Tris-HCl, 150 mmol/l NaCl, 1% [vol./vol.] IGEPAL CA630, one tablet of EDTA-free protease inhibitor cocktail per 50 ml [Roche Diagnostics, Switzerland]) and protein content measured using a Pierce BCA assay (Thermo Fisher Scientific).

#### LC-MS

Samples were extracted and concentrated as previously described [4]. Mouse sample supernatant fractions were analysed after reduction and alkylation by nanoflow LC-MS using a ThermoScientific Ultimate 3000 nano LC system coupled to a Q-Exactive Plus Orbitrap mass spectrometer (ThermoScientific, USA). Relative peptide quantification was performed using Quanbrowser (ThermoScientific) for GLP-1(7–37) and GLP-1(7–36)-amide, which were combined to give ‘total active GLP-1’, INSL5 (A chain), PYY(1–36) and neurotensin by measuring the peptide peak areas in the treated condition compared with the mean of the control-treated cultures. Data were normalised to protein content in primary lysates.

### Statistics

Descriptive statistics (mean ± SEM) were generated using GraphPad Prism (version 7.0, USA). Data were analysed using Student’s *t* test (unpaired and paired, as appropriate), one- and two-way ANOVA with multiple comparisons (Sidak) and ANCOVA, as appropriate. No animals were excluded from the analyses. A *p* value of 0.05 was considered significant.

## Results

**Fig. 1:**
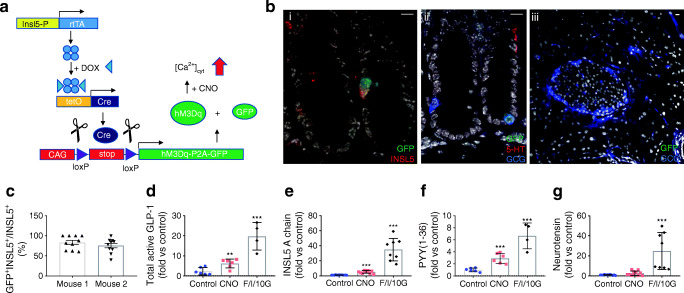
Characterisation of L^distalDq^ model. (**a**) Tetracycline (DOX)-inducible Cre-mediated recombination system specific to distal L cells resulting in DREADD-hM3Dq expression. The rtTA is expressed under the control of the *Insl5* promoter. Induction with DOX (‘TET-On’) drives a Cre-mediated recombination, resulting in the excision of a stop cassette, driving expression of hM3Dq-P2A-Citrine (Citrine is a yellow GFP variant) in distal L cells. (**b**) Representative images from immunohistochemistry-based assessment of Citrine induction in (**i**, **ii**) colonic and (**iii**) pancreatic tissue from DOX-treated Insl5-rtTA×Tet-Cre×Dq mice (**i**) GFP (green)/INSL5 (red), (**ii**) GFP (green)/5-HT (red)/GCG (blue) and (**iii**) GFP (green)/GCG (blue). Scale bars, 10 μm. (**c**) Bars represent percentage of INSL5-positive cells that are positive for GFP (*n* = 2 mice). (**d**-**f**) GLP-1, INSL5, PYY and neurotensin secreted from mouse colonic cultures (*n* = 2–4, in triplicate). Data represent the mean ± SEM of the fold increase of peptide quantification (peak area) of the treated condition compared with the mean of the vehicle treated control triplicates of the same culture. Conditions: control, 10 μmol/l CNO and a combination of forskolin (10 μmol/l) 3-isobutyl-1-methylxanthine (IBMX; 10 μmol/l) and glucose (10 mmol/l) (F/I/10G). **p < 0.01 and ***p < 0.001 vs control by 1-way ANOVA with Dunnett’s post hoc test performed on log_10_-transformed data (vs control). CAG, synthetic promoter composed of cytomegalovirus early enhancer element (C), the promoter, the first exon and the first intron of the chicken beta-actin gene (A) and the splice acceptor of the rabbit beta-globin gene (G); Insl5-P, insulin-like peptide 5 gene promoter; P2A, viral 2a ‘ribosomal stutter’ sequence; stop, stop codon; tetO, tetracycline operator sequence. Circles, vehicle; squares, CNO; diamonds, F/I/10G

We showed previously that INSL5 is produced by the majority of L cells in the distal two-thirds of the mouse colon/rectum [22]. Despite previous reports of Insl5-expression in the hypothalamus [23], we were unable to detect reporter gene expression in the CNS of DOX-induced Insl5rtTAxGCaMP6ΔCMV mice [4], which expressed the reporter in the colon (ESM Fig. 1). To selectively stimulate distal colonic L-cells, we created a new transgenic mouse model in which the reverse tetracycline-controlled transactivator (rtTA) expressed under the control of the *Insl5* promoter drives Cre expression under a TET (tetracycline-controlled transcriptional activation) promoter. Induction with doxycycline drives Cre-mediated recombination, resulting in the excision of a stop cassette, allowing monocistronic expression of hM3Dq, a DREADD (designer-receptor exclusively activated by designer drugs) based on the human M3 muscarinic receptor and the yellow fluorescent protein Citrine from a Cre reporter allele (Fig. 1a; L^distalDq^-cells). Immunostaining of colonic sections from two DOX-treated mice revealed 79% of INSL5-positive cells stained for GFP, detecting Citrine expression in this mouse model (Fig. 1 bi, c). A small number of GFP-positive cells were also positive for 5-HT (<5%), and a small number (<10%) of GCG-positive cells also demonstrated immunoreactivity for 5-HT (Fig. 1bii). In addition, sporadic cells in the epithelium stained positive for GFP (<1%). Hormone secretion from primary mouse colonic cultures from this mouse model was investigated by LC-MS. Cells were stimulated with 10 μmol/l CNO, a pharmacologically inert metabolite of the atypical antipsychotic drug clozapine and ligand for DREADD-Dq [24], which resulted in a significant increase in GLP-1(7–37), INSL5 (A chain) and PYY(1–36) release (Fig. 1d–f). Neurotensin did not respond to treatment with CNO (Fig. 1g).

**Fig. 2:**
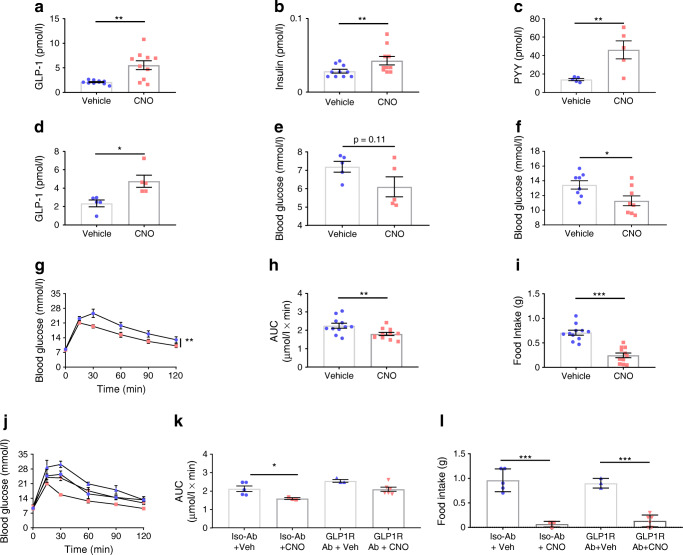
Colonic L cell stimulation improves glucose tolerance, actions directly attributable to GLP-1. (**a**) Plasma GLP-1 (*n* = 10, crossover design), (**b**) insulin [data from same samples as in (**a**)] and (**c**) PYY (*n* = 5, non-crossover design) 15 min post administration of vehicle or CNO (at 0.3 mg/kg i.p.). (**d**) Plasma GLP-1 (*n* = 5, crossover design) and (**e**) corresponding blood glucose 30 min post administration of vehicle or CNO (0.3 mg/kg i.p.). (**f**) Blood glucose in the fed state, 15 min post administration of CNO. (**g**) IPGTT (2 g/kg body weight glucose, administration of vehicle or CNO (at 0.3 mg/kg i.p., delivered contralaterally to glucose). (**h**) AUC and (**i**) 1 h food intake post IPGTT (2 h after administration of glucose). Values presented as group mean ± SEM (*n* = 11, crossover design). Animals were subsequently pre-treated with a GLP1R antibody (GLP1R Ab) or isotype control antibody (Iso-Ab) (**j**) IPGTT (as previous), (**k**) AUC and (**l**) 1 h food intake post IPGTT post administration of vehicle or CNO (0.3 mg/kg i.p., delivered contralaterally to glucose). Values presented as group mean ± SEM (*n* = 5–6 mice per group). *p < 0.05, **p < 0.01 and ***p < 0.001 by unpaired Student’s *t* test (**c**), paired Student’s *t* test (**a**, **b**, **d**, **e**, **f**, **h**, **i**), 1-way ANOVA with Dunnett’s post hoc test (**k**, **l**) or 2-way ANOVA (**g**, **j**). Circles, vehicle; squares, CNO treated; triangles (up), GLP1R ab + vehicle; triangles (down), GLP1R + CNO

In the fasted state, 15 min after in vivo stimulation of L^distalDq^ cells with CNO (0.3 mg/kg i.p.), GLP-1 and PYY plasma levels were 2.7- and 3.3-fold higher than those observed in mice receiving vehicle stimulation, respectively, and there was a small increase in plasma insulin, 15 min post administration, (Fig. 2a-c). Effects on GLP-1 (Fig. 2d) and PYY (vehicle 13.2 ± 2.9 vs CNO 29.2 ± 8.4 pmol/l, *n* = 4–5, *p* < 0.01) were maintained at 30 min. No difference in plasma glucose was observed in the fasting state (Fig. 2e), but in fed mice, stimulation of L^distalDq^ cells significantly reduced blood glucose (*p* < 0.05) (Fig. 2f). Given the hormone profile of the animals and the glucose-lowering effect demonstrated by L^distalDq^ cell stimulation in the fed state, we further explored the effects of L^distalDq^ cell stimulation on glucose tolerance. An IPGTT (2 g/kg body weight), revealed significantly improved glucose tolerance following L^distalDq^ cell stimulation (*p* < 0.01) (Fig. 2g,h). Interestingly, upon refeeding post-IPGTT, food intake was significantly lower following CNO treatment, (*p* < 0.0001) (Fig. 2i). Blockade of GLP1R with a monoclonal antagonistic antibody (GLP1R0017) impaired the CNO-triggered reduction in plasma glucose following an IPGTT (Fig. 2j,k). However, the effect on food intake was maintained (Fig. 2l).

**Fig. 3:**
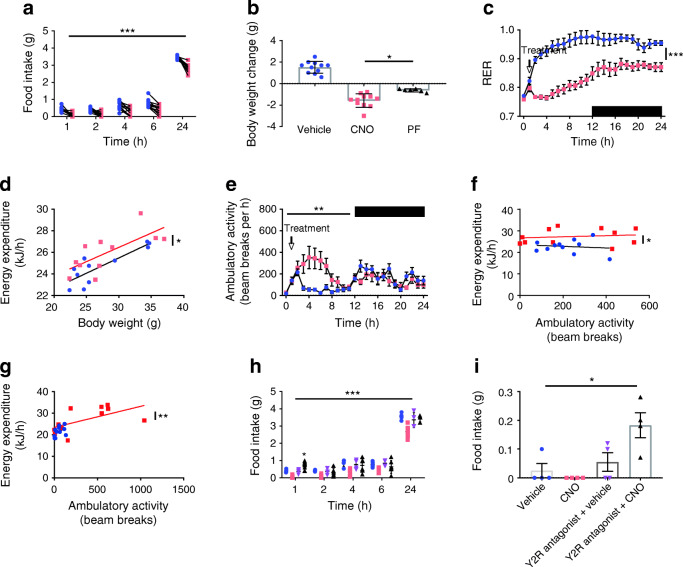
Colonic L cell stimulation reduces food intake, actions directly attributable to PYY, and increases energy expenditure, a consequence of increased activity. (**a**) Food intake post administration of vehicle or CNO (*n* = 11, crossover design). (**b**) Body weight change over the 24 h period, including a pair-fed group (PF) which demonstrate attenuated weight loss. (**c**) RER, (**d**) Energy expenditure, (**e**) ambulatory activity and energy expenditure 1 h (**f**) and 2 h (**g**) post treatment vs ambulatory activity for mice treated with vehicle or CNO (*n* = 11, crossover design). (**h**, **i**) Food intake of animals pre-treated (*t* = −30 min) with JNJ-31020028 (20 mg/kg i.p.) post administration of vehicle or CNO (0.3 mg/kg i.p., delivered contralaterally to vehicle or antagonist pre-treatment, *n* = 4–8 mice per group). Animals in (**h**) had been transiently fasted as in (**a**–**g**) whereas animals in (**i**) had free access to food. Values presented as group mean ± SEM. *p < 0.05, **p < 0.01, ***p < 0.001 by 1-way ANOVA with Dunnett’s post hoc test (**b**, **i**), 2-way ANOVA (**a**, **c**, **e**, **h**) or ANCOVA (**d**, **f**, **g**). Blue symbols, vehicle; Red symbols, CNO, triangles (down), Y2R antagonist + vehicle; triangles (up), Y2R antagonist + CNO. Dark phase is indicated by black bars in **c** and **e**

To further explore this effect, we monitored food intake for 24 h post administration of CNO, which we had previously shown to have no effect on food intake in C57BL/6JN control mice [21]. Following an overnight fast, food intake was significantly reduced following L^distalDq^ cell stimulation (*p* < 0.001) (Fig. 3a). Presumably as a result of the reduced food intake, body weight was significantly reduced (*p* < 0.001) (Fig. 3b). An independent group of animals pair-fed to the treatment group demonstrated attenuated body weight change compared with the CNO group (*p* < 0.05) (Fig. 3b). This suggested an effect on energy expenditure, which was therefore assessed using metabolic cages. To mimic the home-cage data, following an 8 h habitation period, animals were fasted overnight and then re-fed prior to treatment with CNO. Whereas the vehicle-treated group exhibited an increase in RER when food was reintroduced owing to the switch to carbohydrate oxidation, L^distalDq^ cell activation prevented this increase, and this is likely to be because mice continued to oxidise lipid as food intake was suppressed (*p* < 0.001) (Fig. 3c). Energy expenditure and activity levels were higher in the CNO-treated group (Fig. 3d,e and ESM Fig. 2a,b). Higher energy expenditure was evident in the first hour after CNO injection when activity levels were similar (Fig. 3f); by 2 h, the two groups were different both in terms of activity and energy expenditure (Fig. 3g). RER, energy expenditure and activity were unaffected by CNO in C57BL/6JN control mice (ESM Fig. 2c-e).

The effect of CNO on food intake was abolished by pre-treatment with JNJ-31020028 (Fig. 3h), a selective brain-penetrant small molecule antagonist of the neuropeptide Y2 receptor, Y2R [25, 26]. Interestingly, mice that were pre-treated with the Y2R antagonist and then administered CNO ate significantly more than the control group that received vehicle alone 1 h post stimulation of L^distalDq^ cells (Fig. 3h, *p* < 0.05), suggesting that stimulating L^distalDq^ cells might release an orexigenic factor unmasked after blocking Y2R. We repeated this experiment in the fed state, and found that stimulation of L^distalDq^ cells in combination with Y2R antagonism resulted in increased food intake 1 h post administration of CNO (Fig. 3i, *p* < 0.05).

**Fig. 4:**
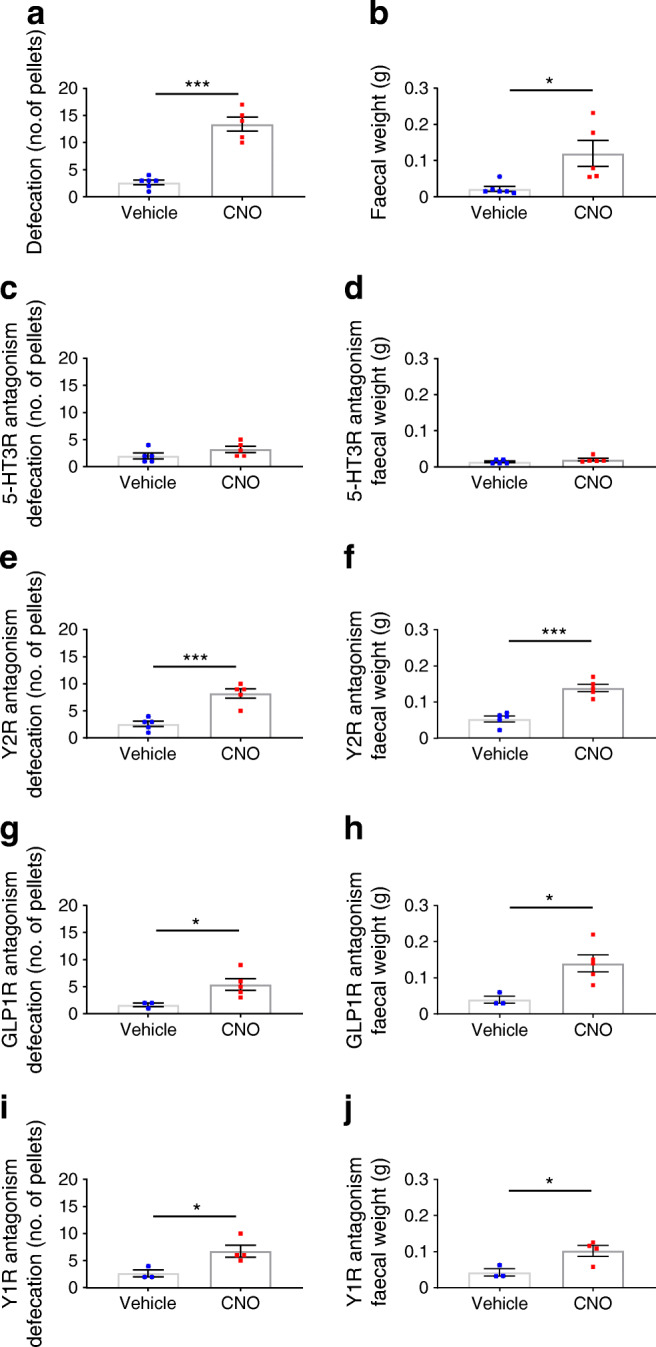
Colonic L cell stimulation increases faecal output, an effect dependent on 5-HT_3_ receptor (5-HT3R) signalling. Defecation quantified as faecal pellet output (**a**, **c**, **e**, **g**, **i**) and faecal weight (**b**, **d**, **f**, **h**, **j**) in response to vehicle or CNO (0.3 mg/kg i.p.) in L^distalDq^ animals (**a**, **b**) without or after pre-treatment with (**c, d**) ondansetron (3 mg/kg i.p.), (**e, f**) Y2R antagonist JNJ-31020028 (20 mg/kg i.p.), (**g**, **h**) Y1R antagonist BIBO3304 (100 μl 0.4 mmol/l i.p.) or (**i**, **j**) GLP1R Ab (GLP1R0017; 19.2 mg/kg s.c.). Values are group mean ± SEM (*n* = 3–5 mice per group, non-crossover design). *p < 0.05, ***p < 0.001 by an unpaired Student’s *t* test. Circles, vehicle; squares, CNO

In addition, we observed that mice exhibited increased faecal output in response to L^distalDq^ cell stimulation, measured either by pellet number or weight (Fig. 4a,b). The effect was abolished by the 5-HT type 3 (5-HT_3_) receptor antagonist ondansetron (Fig. 4c,d) but unaffected by blockage of Y2R (by JNJ-31020028) (Fig. 4e,f), Y1R (BIBO3304) [27] or GLP1R (antagonistic antibody, GLP1R0017) (Fig. 4g–j).

**Fig. 5:**
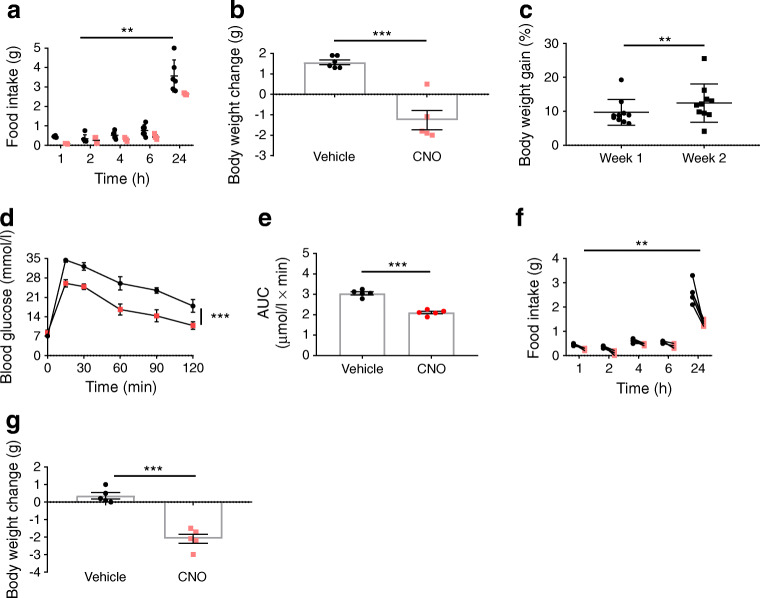
The effects on glucose tolerance and food intake are maintained following exposure to HFD. (**a**) Food intake and (**b**) body weight change (in g) following initial exposure to an HFD (*n* = 5–6 mice per group). (**c**) Body weight gain (in %) following 2 weeks of exposure to an HFD. (**d**) IPGTT (2 g/kg glucose), (**e**) AUC post administration of vehicle or CNO (0.3 mg/kg i.p., delivered contralaterally to glucose, *n* = 5, crossover design). (**f**) Food intake and (**g**) body weight change post administration of vehicle or CNO (0.3 mg/kg i.p., *n* = 5, crossover design). Values are group mean ± SEM. **p < 0.01, ***p < 0.001 by unpaired Student’s *t* test (**b**), paired Student’s *t* test (**c**, **e**, **g**) or 2-way ANOVA (**a**, **d**, **f**). Circles, vehicle; squares, CNO

Initial exposure to an HFD generally results in hyperphagia. Stimulating L^distalDq^ cells at the time of transfer to an HFD reduced food intake and body weight change (*p* < 0.01 and *p* < 0.001, respectively) (Fig. 5a,b). Exposure to an HFD (2 weeks) significantly increased body weight (*p* < 0.01) (Fig. 5c). Activation of L^distalDq^ cells after 2 weeks on an HFD significantly improved glucose tolerance (blood glucose levels and AUC, as assessed by IPGTT) compared with vehicle (*p* < 0.001 and *p* < 0.0001, respectively) (Fig. 5d,e). Under HFD conditions, activation of L^distalDq^ cells also reduced food intake and body weight (*p* < 0.01 and *p* < 0.001, respectively) (Fig. 5f,g), mirroring observations in chow-fed mice.

## Discussion

Utilising a novel INSL5-Dq-DREADD mouse model, this study verifies the colon as an endocrine organ, as products from distal L cells were detected in the circulation and affected glucose tolerance, food intake and body weight. CNO-triggered stimulation of INSL5^+^ L cells in primary colonic cultures triggered secretion of GLP-1, INSL5 and PYY, and in vivo increased plasma GLP-1 and PYY, improved glucose tolerance and reduced food intake.

The concept of GLP-1 as a gut-derived incretin hormone is not new but is currently controversial, as recent publications have identified the pancreas as a potential alternative source of GLP-1 [15, 16, 28]. Original reports had identified the intestine as the site of bioactive GLP-1 production, whereas pancreatic extracts and perfusates contained very little [29, 30]. Pancreatic alpha cells were also shown to normally process proglucagon to generate N-terminally extended inactive GLP-1 peptides [31]. Plasma GLP-1 levels increase following oral glucose or meal ingestion, but not after intravenous glucose administration, suggesting the circulating peptide is largely gut-derived [32], whereas islet GLP-1 production has largely been reported in models of inflammation and diabetes [17, 33]. However, reactivation of *Gcg* expression in the pancreas (using a pancreatic and duodenal homeobox-1 [*Pdx1*] promoter), but not the intestine (using a villin [*Vil1*] promoter) in a *Gcg* knockout background had an effect on insulin secretion sensitive to the GLP1R blocker exendin-9–39 [15], leading the authors to conclude an important role for pancreatic GLP-1. Although paracrine GLP-1-mediated signalling from pancreatic alpha to beta cells was proposed, it was subsequently shown that glucagon, present at much higher concentrations than active GLP-1 in the pancreas, is likely to dominate any intra-islet GLP1R-dependent stimulation of pancreatic beta cells [30, 34–36]. By default, a mechanism entirely dependent on intra-islet crosstalk cannot underlie the incretin effect, as islets require a signal additional to a plasma glucose rise to recognise if the glucose arises from intestinal absorption; for intra-islet crosstalk to participate in the incretin effect, this additional information would have to be supplied by another means, such as autonomic nervous signals initiated by the intestine. Despite previous reports of INSL5-positive hypothalamic neurons [23], we were unable to demonstrate GFP-immunostaining in the CNS of Insl5-rtTA×GCaMP6f mice. Whilst this might be due to limited DOX penetration of the CNS [37], together with the absence of detectable GFP signals in the pancreas of Insl5-rtTAxTet-CrexDq mice (Fig. 1biii), this strongly supports a peripheral, intestinal origin of the observed effects on glucose homeostasis and feeding in response to CNO. Our data thus demonstrate that direct colonic L cell stimulation improves glucose tolerance via GLP-1, as the effect was blocked by a GLP1R antagonistic antibody. Given the scarcity of vagal innervation of the distal colon, an afferent signal triggering a vagal reflex to the pancreas and release of pancreatic GLP1R-agonist seems unlikely, and a classical endocrine signalling pathway from the gut to the pancreas seems the most probable explanation for our findings.

The inhibition of food intake triggered by L^distalDq^ cell stimulation was not prevented by GLP1R antagonism, but instead seemed attributable to PYY, as it was abolished by the Y2R inhibitor JNJ-31020028. Surprisingly, Y2R antagonism unmasked an orexigenic effect of stimulating colonic L cells in the fasted and fed state, which we speculate may be attributable to INSL5. Unfortunately, we are unable to measure plasma INSL5 in mice, but we demonstrated that INSL5 was co-released with PYY and GLP-1 by CNO-mediated stimulation of L^distalDq^ cells in vitro, consistent with our previous report that all three peptides are co-packaged into the same vesicular pool in these cells [4]. In the absence of Y2R antagonism, however, the anorexigenic effect of PYY dominated the effect of L^distalDq^ cell activation on food intake, suggesting that any low-level orexigenic activity of L cell-released peptides is physiologically unimportant. Further investigation into the role of colonic INSL5 is, nevertheless, warranted given the mixed reports that it exhibits orexigenic activity [3], modulates hepatic glucose production [38] and/or stimulates insulin secretion [39]. Although we also here observe a possible orexigenic effect of INSL5, others have failed to observe effects on food intake even at pharmacological doses of chemically synthesised INSL5 (native and pegylated forms) [40].

Interestingly, the reduced body weight observed following L^distalDq^ cell stimulation seems to be a consequence not only of reduced food intake, but also of increased energy expenditure, as demonstrated by our pair-fed group and studies in metabolic cages. The difference in RER between the CNO and vehicle groups appears profound but is likely to reflect food intake, with control mice switching to carbohydrate oxidation as they re-feed on chow following the overnight fast, whereas the CNO group continued to burn fat as food intake was suppressed. The explanation for the higher activity and energy expenditure of CNO-treated mice remains unclear and seems unlikely to be attributable to GLP-1 or PYY, which have been characterised extensively in the literature. Whilst we did not formally test whether GLP1R or Y2R blockade prevented these effects using metabolic cages, visual observations of mice treated with GLP1R antibody or JNJ-31020028 prior to CNO injection suggested that increased locomotor activity was still observed in the presence of these inhibitors. Oxyntomodulin, which is co-produced with GLP-1 from the proglucagon peptide in L cells, has been shown to increase energy expenditure, likely through its activity on the glucagon receptor, although it was not found to increase locomotor activity [41, 42].

L^distalDq^ cell stimulation increased faecal weight and pellet number; effects that were abolished by pre-treatment with ondansetron, an inhibitor of 5-HT_3_ receptors. Both 5-HT_3_ and 5-HT_4_ receptors have been implicated previously in regulating faecal output in mice [43, 44]. Studies in mice lacking *Tph1*, the enzyme responsible for mucosal 5-HT production, also concluded that enterochromaffin cells are important for propagation of colonic migrating motor complexes and pellet propulsion [45]. Our results suggest that enterochromaffin cells were activated by L^distalDq^ cell stimulation. Direct activation of enterochromaffin cells seems unlikely, as only a small number of GFP-labelled cells stained positive for 5-HT, consistent with previous reports of only a small overlap of *Gcg* and *Tph1*-expressing cells in the small intestine [46] and colon [22]. Although we cannot exclude that direct CNO stimulation of rare cells positive for both INSL5 and 5-HT might have been sufficient to activate faecal expulsion, we speculate that L^distalDq^ cell stimulation indirectly activated enterochromaffin cells via paracrine crosstalk. Enterochromaffin cells have been shown previously to express *Glp1r* [47], but antagonism of GLP1R did not prevent the increased faecal output in our model. Other possible enterochromaffin targets for L cell products include Y1R and relaxin/insulin-like family peptide receptor 4 (RXFP4), the receptor for INSL5, mRNA for which we found to be expressed in colonic *Tph1*-positive cells [22]. Both Y1R and RXFP4 are predominantly G_i_-coupled, but pertussis toxin-sensitive stimulation of 5-HT release has been shown downstream of α_2_-adrenergic stimulation [48]. However, Y1R inhibition had no effect on accelerated faecal output in our model, consistent with NPY and/or PYY knockout resulting in increased rather than decreased faecal output in response to restraint-stress [27, 49], despite an apparent inhibition of colonic transit by Y1R inhibition in vitro [27].

### Conclusions and outlook

The physiological roles of colonic L cells, which contain a large proportion of the body’s endogenous reserves of GLP-1 and PYY, still remain a mystery, but our results indicate they would be good targets for small molecules aiming to increase GLP-1 and PYY release for the treatment of diabetes and obesity. Supporting this idea, ingestion of propionate ester, which stimulates release of GLP-1 and PYY from colonic L cells via the SCFA receptor known as free fatty acid receptor 2 (FFAR2), reduced energy intake and weight gain in humans [50], and rectal administration of bile acids in human volunteers also decreased blood glucose and food intake, and it is likely that this occurs via G-protein-coupled bile acid receptor 1 (GPBAR1) [51]. Colonic L cells are enriched for a variety of G-protein-coupled receptors, but although it would be tempting to speculate that these might be targetable with non-absorbable gut-restricted small molecules, several of the receptors seem to be located basolaterally on L cells [12, 52, 53], requiring local drug absorption. It will be particularly interesting to discover whether maximal pharmacological stimulation of colonic L cells in humans is capable of reproducing the very high postprandial GLP-1 and PYY levels observed after bariatric surgery, and whether stimulating colonic L cells can mimic the beneficial effects of surgery on glucose metabolism and body weight.

## Electronic supplementary material

ESM 1 (PDF 656 kb)

## Data Availability

Data are available on request from the authors.

## Abbreviations

CNO: Clozapine *N*-oxide
DOX: Doxycycline
DREADD: Designer Receptors Exclusively Activated by Designer Drugs
EEC: Enteroendocrine cell
GLP: Glucagon-like peptide
GLP1R: Glucagon-like peptide-1 receptor
HFD: High-fat diet
hM3Dq: DREADD-receptor coupling to Gq derived from human muscarinic receptor 3
5-HT: 5-Hydroxytryptamine (serotonin)
5-HT_3_: 5-Hydroxytryptamine (serotonin) type 3 (receptor)
INSL5: Insulin-like peptide-5
L^distalDq^: Distal colonic L cell expressing hM3Dq
PYY: Peptide YY
RER: Respiratory exchange ratio
rtTA: Reverse tetracycline-controlled transactivator
SCFA: Short-chain fatty acid
Tet-on: Tetracycline-activated expression
Y1R: Neuropeptide Y receptor type 1
Y2R: Neuropeptide Y receptor type 2
