# Supplementary material for: Selective stimulation of colonic L cells improves metabolic outcomes in mice
Source: Diabetologia. 2020 Apr 27;63(7):1396–407. doi: 10.1007/s00125-020-05149-w (PMC7286941; doi:10.1007/s00125-020-05149-w)
Supplement: Supplementary file 1 — (PDF 656 kb) [file 125_2020_5149_MOESM1_ESM.pdf]

ESM Table 1.

Target (cat. No)	Primary	Secondary (dilution 1:300, all ThermoFisher Scientific)
INSL5	1:500 (rat; Takeda, Japan)	Donkey anti-rat conjugated with AlexaFluor 488 (A32790)
5-HT (20080)	1:1000 (rabbit; Immunostar, USA)	Donkey anti-rabbit conjugated with AlexaFluor 550 (A32794)
GCG (sc-514592)	1:50 (mouse; Santa-Cruz Biotechnologies, USA)	Donkey anti-mouse conjugated with AlexaFluor 555 (A32773)
GFP (ab5450)	1:1000 (goat; Abcam, UK)	Donkey anti-goat conjugated with AlexaFluor647 (A32849)

ESM Table 1 Antibody reagents

Hoechst nuclear stain (1:1000) was also used in immunohistochemistry experiments

ESM Fig. 1.

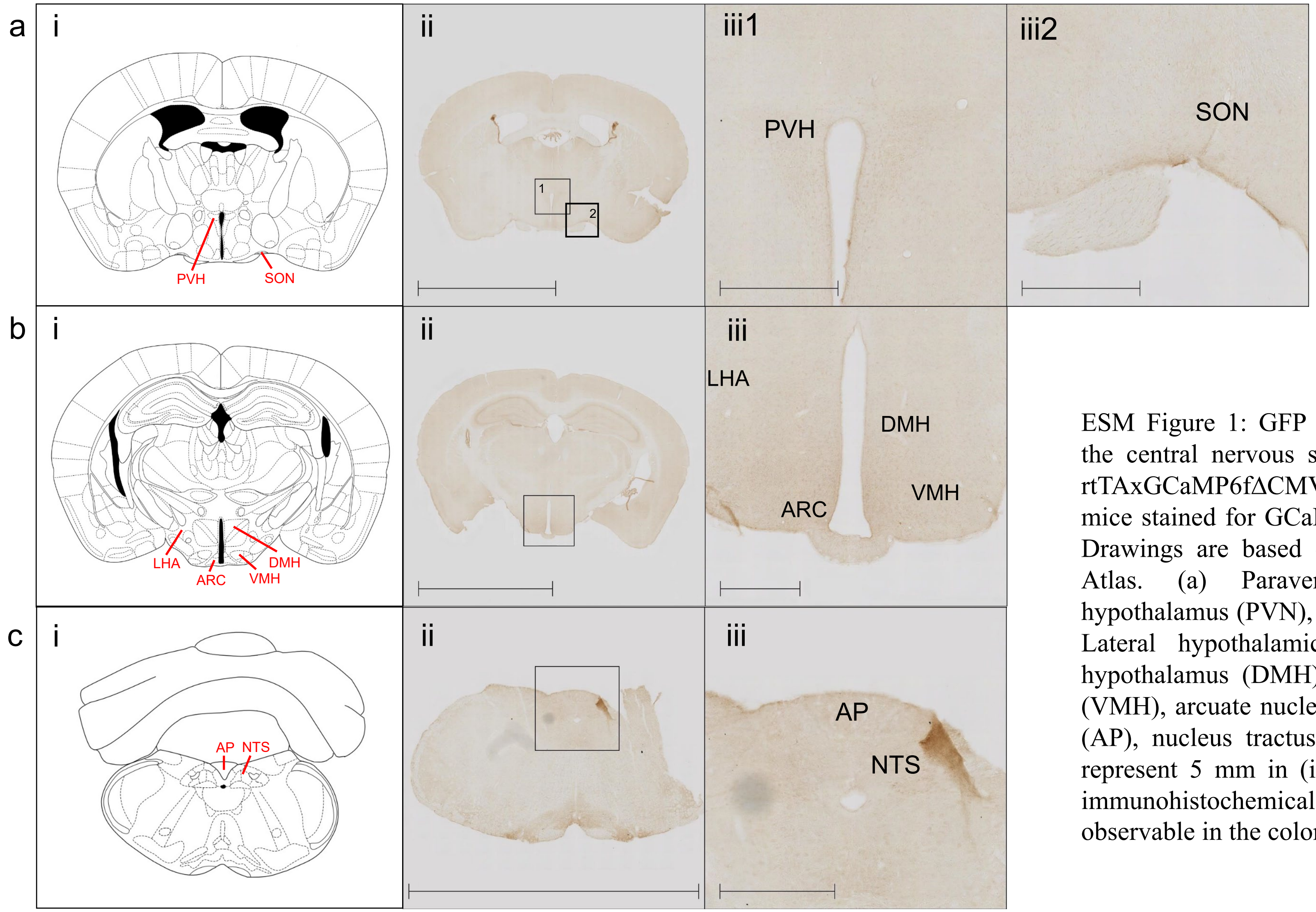

ESM Figure 1: GFP expression was undetected in the central nervous system of Dox-induced Insl5-rtTAxGCaMP6fΔCMV mice. Coronal sections from mice stained for GCaMP6f (using a GFP antibody). Drawings are based on the Paxinos Mouse Brain Atlas. (a) Paraventricular nucleus of the hypothalamus (PVN), supraoptic nucleus (SON). (b) Lateral hypothalamic area (LHA), dorsomedial hypothalamus (DMH), ventromedial hypothalamus (VMH), arcuate nucleus (ARC). (c) Area postrema (AP), nucleus tractus solitarius (NTS). Scale bars represent 5 mm in (ii) and 500 µm in (iii). GFP-immunohistochemical staining was readily observable in the colon of these mice (not shown).

ESM Fig. 2.

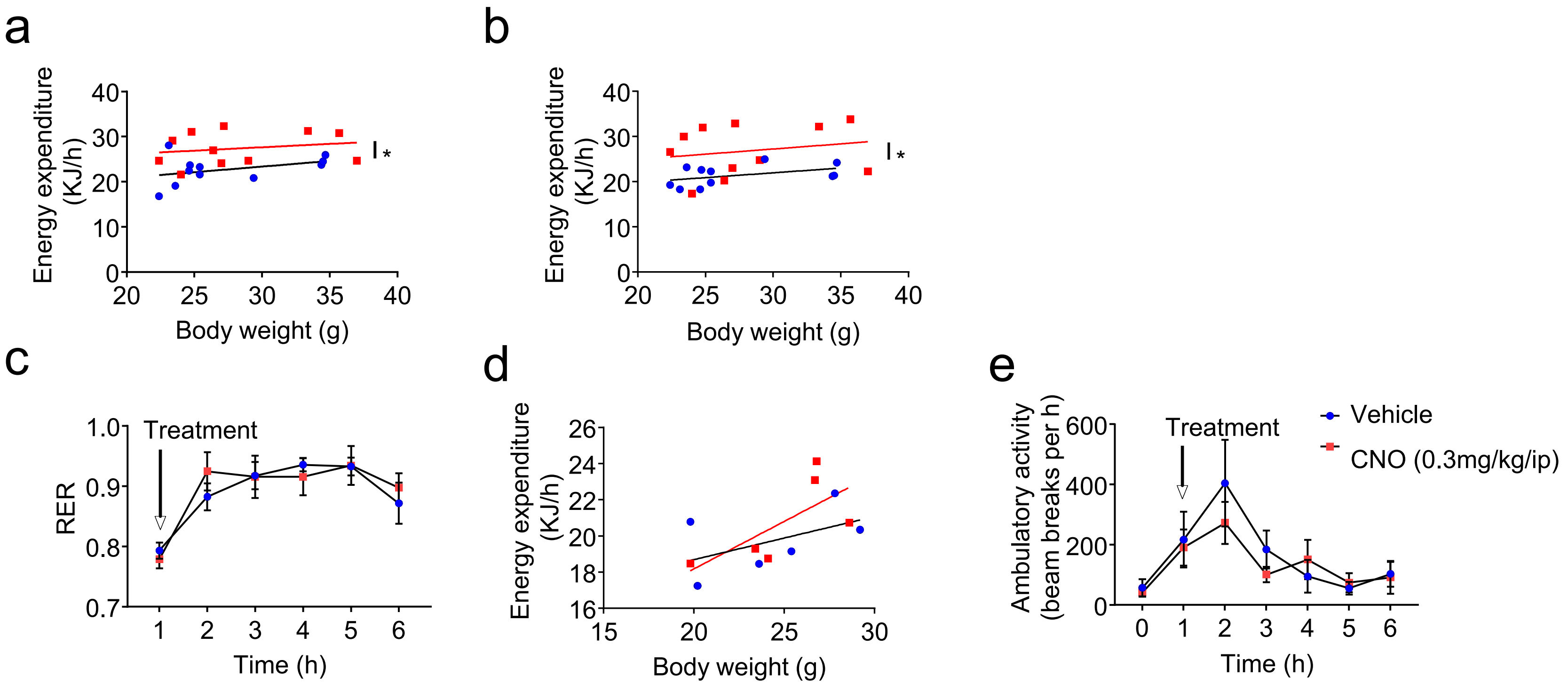

ESM Figure 2: Energy expenditure is increased by colonic L cell stimulation. (a) Energy expenditure 1h and (b) 2 h post administration of CNO in Dox-induced Insl5-rtTAxTet-CrexDq mice. Values are group mean ± SEM (n = 11, crossover design). * p < 0.05 by ANCOVA (a, b). (c) RER, (d) energy expenditure and (e) activity were unaffected by CNO in control C57Bl/6JN mice. Values are group mean ± SEM (n = 6 mice per group, non-crossover design).
